# Molecular identification of *Ehrlichia*, *Anaplasma*, *Babesia* and *Theileria* in African elephants and their ticks

**DOI:** 10.1371/journal.pone.0226083

**Published:** 2019-12-05

**Authors:** Edward King’ori, Vincent Obanda, Patrick I. Chiyo, Ramon C. Soriguer, Patrocinio Morrondo, Samer Angelone

**Affiliations:** 1 Department of Animal Science, University of Santiago de Compostela, Santiago, Spain; 2 Veterinary Department, Kenya Wildlife Service, Nairobi, Kenya; 3 Institute of Primates Research, National Museums of Kenya, Nairobi, Kenya; 4 Estación Biológica de Doñana, Consejo Superior de Investigaciones Científicas (CSIC), Sevilla, Spain; 5 Institute of Evolutionary Biology and Environmental Studies (IEU), University of Zurich, Zurich, Switzerland; University of Kentucky College of Medicine, UNITED STATES

## Abstract

Although historical records indicate the presence of *Ehrlichia* and *Babesia* in African elephants, not much is known about their prevalence and diversity in elephants and their ticks, *Amblyomma thollonii* and *Rhipicephalus humeralis*. We amplified and sequenced the hypervariable V4 region of the 18S rRNA gene of *Babesia* and *Theileria* and the heat shock protein gene (groEL) of *Ehrlichia*/*Anaplasma* in DNA extracted from elephant blood (n = 104) and from elephant ticks (n = 52). Our results showed that the African elephants were infected with a novel *Babesia* spp. while *A*. *thollonii* was infected with *Theileria bicornis* and *Theileria* cf. *velifera*. This is the first record of *T*. *bicornis*; a protozoan that is linked to fatal infection in rhinoceros in a tick. Elephants and their ticks were all infected with a species of *Ehrlichia* like that identified in Japanese deer. The prevalence of *Babesia* spp., *Theileria* spp. and *Ehrlichia* spp. in ticks was higher than that of their elephant hosts. About 13.5% of elephants were positive for *Theileria* or *Babesia* while 51% of *A*. *thollonii* ticks and 27% of *R*. *humeralis* ticks were positive for *Theileria* or *Babesia*. Moreover, 5.8% of elephants were positive for *Ehrlichia or Anaplasma* compared to 19.5% in *A*. *thollonii* and 18% in *R*. *humeralis*. There was no association between the positive result in ticks and that of their elephant hosts for either *Babesia* spp., *Theileria* spp. or *Ehrlichia* spp. Our study reveals that the African elephants are naturally infected with *Babesia* spp and *Ehrlichia* spp and opens up an opportunity for further studies to determine the role of elephant as reservoirs of tick-borne pathogens, and to investigate their potential in spreading these pathogens as they range extensively. The presence of *T*. *bicornis* in *A*. *thollonii* also suggests a need for experiments to confirm its vector competence.

## Background

Wildlife species harbor several important tick-borne hemoparasites such as *Theileria*, *Babesia*, *Ehrlichia* and *Anaplasma* where they may occur as asymptomatic infections. However, asymptomatic infections may progress to clinical disease when the host is exposed to ecological stressors such as co-infections with other pathogens [1], translocation [2, 3], malnutrition [4] and drought. Clinical disease may increase overall morbidity and mortality, reduce fecundity and infant survival, factors that influence population performance and increases extinction risk for small endangered wildlife populations. For example co-infection of Canine Distemper Virus with *Babesia* species in lions has been documented to cause lethal disease characterized by higher mortality in lions [1]. In rhinoceros and several species of antelopes, mortality associated with *Theileria* and *Babesia* infections has been shown to occur following capture and translocation [2, 5]. There are also cases where infections by *Theileria* in synergy with malnutrition have been implicated as a cause for high calf mortality and population decline in the roan and sable antelopes in South Africa [4, 6].

The African elephant, like other wildlife species, is infested by various species of ticks that are known to be competent vectors of many blood-borne pathogens. *Amblyomma thollonii* and *Rhipicephalus humeralis* are two species of ticks mainly found as adult stages in the African elephant and other pachyderms, including the rhinoceros, and hippos [7–10]. These ticks are found in elephants in Kenya and other elephant ranges in Africa [9–13]. The two species of ticks are three-host ticks; immature stages are less host-specific and parasitize on domestic ruminants such as cattle, sheep and goats [14, 15] whereas the adults parasitize predominantly elephants and sometimes also found on rhinoceros which are considered an alternative host [16, 17] as well as the giant forest hog and warthog [13]. The ticks that commonly feed on elephants are known, yet the hemoparasites they transmit to the African elephants are not well described [7–9]. Previously, unknown species of *Babesia* were examined using microscopy in an African elephant in Kenya [18] and since then, no further publication by morphology or molecular techniques has been advanced.

The genera *Ehrlichia* and *Anaplasma* belong to the family Anaplasmataceae (Order Rickettsiales) and comprise of diverse species that infect and cause disease in a wide range of wild and domestic animals including humans and they are associated with an emerging group of zoonotic diseases. *Rickettsia* sp. Uilenburgi strain [19] have been detected in elephants while *Ehrlichia ruminantium* (formerly *Cowdria ruminantium*), was linked to cowdriosis (Heartwater) disease in the African elephant [20]. Further, an experimental transmission of *Ehrlichia* spp. by *A*. *thollonii*, suggest that this elephant bont-tick is possibly among the species of *Amblyomma* ticks that transmit Heartwater disease.

In this study, molecular tools are used to identify and characterize infection of protozoans in the genera *Babesia* and *Theileria* (Order Piroplasmidae), commonly referred to as piroplasm and bacteria in the genera *Ehrlichia* and *Anaplasma* (Order Rickettsiales) in both the host blood (Elephant) and ticks collected from the same individual host. This approach links the parasites to the vector and host, specifically revealing the parasite sharing, transmission and infection dynamics in less studied host-vector systems like the African elephant. This information is important in understanding the epidemiology of these parasites and especially the roles of these ticks in disease maintenance and spread.

## Materials and methods

### Ethics statement

This study was permitted by the Research and Ethics Committee of the Kenya Wildlife Service (KWS/BRM/5001), the Institution mandated to protect and conserve Wildlife in Kenya. Blood samples were collected during scheduled interventions (clinical treatment and translocations) and involved experienced wildlife veterinarians who followed the approved protocols and guidelines on Wildlife Veterinary Practice 2006 and the Veterinary Surgeons Act Cap 366 of the Laws of Kenya that regulates veterinary practices in Kenya.

### Study area

**Fig 1:**
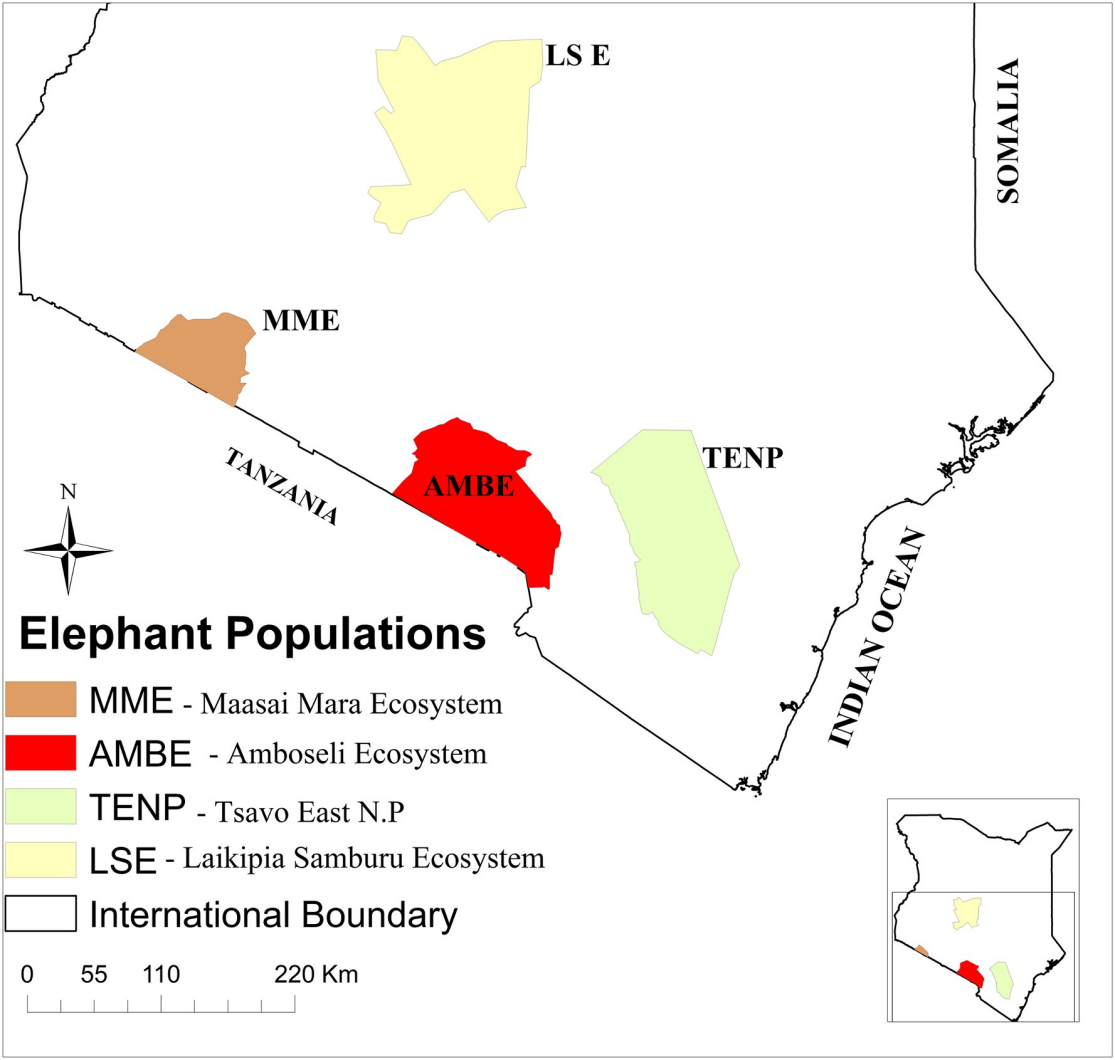
Map of Kenya showing locations of the sampled populations of the African elephant and their ticks.

We conducted our study in four major ecosystems that sustain more than 50% of Kenya’s elephant population. The locations include the Mara-Serengeti Ecosystem, the Amboseli Ecosystem (AMBE) and the Tsavo Ecosystem in southern Kenya and Laikipia-Samburu Ecosystem (LSE) in north central Kenya (Fig 1). The MSE which lies between longitude 34° and 36° E and latitude 1° and 2° S and covers 25,000 km^2^ consist of the Maasai Mara National Reserve and adjacent wildlife conservancies in Kenya and the Serengeti National Park and associated game reserves in Tanzania. In this ecosystem, we focused on the Maasai Mara National Reserve and associated wildlife conservancies covering some 7000 Km^2^, hereafter -Maasai Mara Ecosystem (MME). Its annual rainfall ranges from 650mm in the south east to 1300mm in the north west [21] and vegetation in the ecosystem is dominantly grassland with scanty cover of shrubs and thorny bushes [20]. The Amboseli ecosystem (AMBE) covers 8500 km^2^ and lies between longitude 36.75° and 38° E and latitudes 3° N and 3° S (Fig 1) comprising Amboseli National Park, Chyulu National Park and adjacent group ranches and community wildlife conservancies. The ecosystem receives an average rainfall of 341–890 mm per annum and the area is characterized by swamps, wooded bushlands, open woodlands, and open grasslands. Tsavo Ecosystem lies between 2–4°S, and 37.5–39.5°E and covers about 48,319 km^2^ including Tsavo East National Park, Tsavo West National Park, South Kitui National Reserve and adjacent ranches and wildlife conservancies. Our sampling mainly focused on Tsavo East National Park (TENP) which covers an area approximately 13,000 km^2^ and receives an annual rainfall that averages 300–600 mm per year and the vegetation is characterized by riverine formations, acacia grassland and mixed bushland [22]. The Laikipia Samburu Ecosystem (LSE) lies between longitude 36–38.5 E and latitude 0–2.5 N and encompasses an area of 33,817 km^2^ consisting of Samburu, Buffalo Springs and Shaba National Reserves. LSE also includes several community and private wildlife conservancies including Ol Pejeta, Ol Jogi, Lewa, Solio Conservancies, Namunyak, Kalama, Meibae and Il Ngwesi. LSE receives an annual rainfall varying between 300–1250 mm per annum [23]. Vegetation cover is very diverse in this ecosystem and range from the lowland, xeric *Acacia* and *Commiphora* and scrub bush lands to the highland, mesic cedar and camphor forests.

These ecosystems are major strongholds of the Kenya’s elephant population. Elephant total counts conducted between 2017 and 2018 show that the Maasai Mara and adjacent wildlife conservancies supports 2,493 elephants [24], the Amboseli Ecosystem 2,127 elephants [25] and Tsavo East National park 7,727 elephants [26]. The Tsavo ecosystem has 14,000, the largest elephant population in the country. LSE has 7,166 elephants [27] the second largest population of elephants in Kenya after the Tsavo ecosystem [28].

### Sampling elephants for blood

The elephants were chemically immobilized for management interventions and other studies using Etorphine hydrochloride (Wildlife Pharmaceuticals (PTY) ltd) at a dosage of 14mg or 18mg for adult female and male respectively. A total of 104 blood samples were collected which consisted of 48 elephants from Tsavo East National Park, 28 elephants from the Laikipia Samburu Ecosystem, 20 elephants from the Maasai Mara National Reserve, and 8 from the Amboseli National Park. Blood (~10ml) was taken from the ear vein of the elephants was placed in an EDTA tube and 2ml aliquoted into cryovials and preserved in liquid Nitrogen while in the field and maintained frozen in a -40°C Freezer until analysis.

### Tick sampling and identification

Engorged adult ticks were dislodged off from the ear and trunk of immobilized elephants. Ticks were collected from most individuals that were sampled for blood. The ticks from the same elephant were placed into a single cryovial. The ticks were preserved frozen in liquid nitrogen pending identification and analysis. Ticks were identified using morphological characteristics following published tick identification keys [10, 17, 29]. We focused mainly on the identification of *Amblyomma tholloni* and *Rhipcephalus humeralis* because we were interested in examining the hemoparasites they carry being ticks commonly infesting elephants.

### DNA extraction from elephant blood and ticks

DNA was extracted from 200 μl EDTA of elephant blood using DNeasy blood and tissue kit (QIAGEN, Hilden, Germany) as described by the manufacturer. Each tick of the 52 ticks, (41 *A*. *thollonii* and 11 *R*. *humeralis*), were processed individually. Each tick was placed in 1.5 ml tube and immersed under liquid nitrogen to freeze-dry and following ground into powder using pellet pestles (Sigma Aldrich, Missouri, USA). The powder was then homogenized in 360 μl PBS buffer (pH = 7.4) and vortexed for 90 seconds. Extraction of total nucleic acids was carried out from 200 μl of the homogenate using DNeasy blood and tissue kit (QIAGEN, Hilden, Germany) following the manufacturer’s instructions. Extracted DNA was then used in polymerase chain reaction (PCR).

### *Theileria* and *Babesia* PCR amplification

Nested PCR targeting the 18S rRNA gene segment of *Theileria* and *Babesia* parasites from genomic DNA of elephant blood and ticks. Primary amplification of the target genes was carried out using ILO-9029 (5’CGGTAATTCCAGCTCCAATAGCGT-3’) and ILO-9030 (5’- TTTCTCTCAAAGGTGCTGAAGGAGT-3’) primer sets while the secondary amplifications were done using ILO-9029 (5’CGGTAATTCCAGCTCCAATAGCGT-3’) and ILO-7782 (5’-AACTGACGACCTCCAATCTCTAGTC -3’) primer sets as described by [30]. The oligonucleotides used in this study were synthesized at Macrogen Inc., Europe. Primary PCR reactions were carried out in a 10 μl total volume that consisted of 5 μl HotStarTaq Master Mix (QIAGEN, Hilden, Germany), 0.5 μl of 10 pmol/ul of each forward and reverse primers, 2 μl of the DNA template and 2 μl of sterile PCR water. The cycling conditions included an initial denaturation at 95°C for 15 minutes, followed by 30 cycles (95°C for 30s, 55°C for 30s, and an extension at 72°C for 1 minute) and a final extension at 72 °C for 5 minutes in a T100 thermal cycler (Bio-Rad). The secondary PCR reaction volume was 25 μl consisting of 0.5 μl each of both primers, 1.0 μl of the primary PCR product, 10.5 μl of sterile PCR water and 12.5 μl of HotStarTaq Master Mix (QIAGEN, Hilden, Germany). The cycling conditions used were identical to those of the primary PCR. The positive control was a *Theileria parva* positive sample from previous work in the laboratory while sterile PCR water was used as a negative control for the amplifications. The amplicons were resolved alongside a Gelpilot 100bp plus ladder (QIAGEN, Hilden, Germany) in a 1.5% agarose gel stained with ethidium bromide and run 90 volts for 35 minutes before visualization under UV illumination. The size of the PCR product was about 450–500 bp.

### *Ehrlichia/Anaplasma* PCR amplification

A nested PCR amplification targeting the Heat shock protein gene (*groEL* gene) of the *Anaplasmataceae*, was undertaken as described by Park et al [31]. GroEL is the highly conserved heat shock chaperonin protein used in phylogenetic relationship of bacteria and is better at differentiating *Ehrlichia* and *Anaplasma* species similar to the groESL gene consisting of 1,200 bp despite its short sequence (200–300 bp) [31]. Primary amplification was carried out using EF1 (5'- CTGAYGGTATGCAGTTTG-3'and ER2 (5'- AYRYYTTTAGCAGTACC-3') primer sets while secondary amplification was done using EF3 (5'- GGTATGCAGTTTGAYCG-3') and ER4 (5'- TCTTTTCTYCTRTCACC-3') primer sets. Primary amplification was done in a 10 μl total reaction volume that consisted 1 μl template DNA, 0.5 μl of 10pmol/ μl of each primer, 5 μl OneTaq^®^ Quick-Load^®^ 2X Master Mix with Standard Buffer (New England Biolabs-NEB, Massachusetts, USA), and 2 μl nuclease free PCR water. The primary amplification conditions included an initial denaturation at 94 for 1 minute followed by 20 cycles of denaturation at 94 for 20s, annealing at 50 for 20 sec and extension at 68 for 30s, followed by a final extension at 68 for 5 minutes in a T100 thermal cycler (Bio-Rad). The secondary PCR total reaction volume was 25 μl consisting of 0.5 μl each of both primers, 1.0 μl of the primary PCR product, 10.5 μl of sterile PCR water and 12.5 μl of HotStarTaq Master Mix (QIAGEN, Hilden, Germany). A positive *Anaplasma spp* sample from other work in the lab was used as a positive control while sterile PCR water was used as a negative control for the amplifications. The conditions of the nested PCR were the same as those of the primary PCR. The PCR products, 300 bp in size, were visualized in a similar procedure as products of *Babesia*/*Theileria*.

### Sequencing, editing, pathogen identification and phylogenetic analyses

All positive PCR products were purified and sequenced at Macrogen Inc., Europe in both the forward and reverse directions. Chromatograms for the forward and reverse sequences aligned and edited using SeqTrace and the poor-quality sequences were discarded [32]. The consensus nucleotide sequences were aligned using MUSCLE v. 3.8.31 [33] algorithm in the MEGA X software. Unique sequences, herein referred to as haplotypes were identified from aligned sequences using DnaSP v 5.10.01 [34]. Sequences with the highest similarity to our haplotypes were identified from GenBank [35] using the BLASTn algorithm [36]. In order to classify our haplotypes into species or clusters of species, at least two representative sequences of each known *Theileria*, *Babesia*, *Ehrlichia* and *Anaplasma* species from GenBank were obtained and combined to corresponding reference/matching sequences from GenBank for phylogenetic analyses. All edited *Theileria* and *Babesia* sequences from this study were deposited in GenBank with accession number MN595045-MN595058 and all sequences for *Ehrlichia* were deposited in GenBank with accession numbers, MN602332-MN602336.

All these sequences were aligned using MUSCLE v. 3.8.31 [33] in MEGA X separately for *Theileria*, *Babesia*, and *Ehrlichia/Anaplasma*. The best model of sequence evolution and rate heterogeneity for the aligned genus specific sequences was estimated using MEGA X [37]. For *Babesia* and *Theileria* phylogenies, the best fit nucleotide substitution model was the Kimura 2-parameter model [38] with a discrete Gamma distribution to model evolutionary rate differences among sites (*Babesia*; G = 0.469, *Theileria*, G = 0.204). The rate variation for some sites was allowed to be evolutionarily invariable in both *Babesia* (I = 34.6% sites) and *Theileria* (I = 47.1% of site) sequence evolution models. *Ehrlichia*’s best model for nucleotide evolution was Tamura 3-parameter model [39] with a discrete Gamma distribution for evolutionary rate differences among sites (5 categories (+G, parameter = 0.209)).

Phylogenetic relationships among *Babesia*, *Theileria* and *Ehrlichia* sequences were carried out using maximum likelihood analyses (MEGA X) and the evolutionary models and parameters stated above. The consensus tree topologies were evaluated for statistical support of internal tree branches using 1000 bootstrap iterations [40]. Nucleotide divergence or the average number of nucleotide substitutions per site between haplotypes from this study and sequences from known species from GenBank was estimated using the Jukes and Cantor model.

The statistical variation in prevalence of infection across populations and between hosts (ticks versus elephants) was evaluated using Chi-square tests performed using the R software for statistical computing [41].

## Results

Out of the 104 genomic DNA samples from Kenyan elephants amplified, only 13.5% (14/104) were positive for *Theileria* or *Babesia* based on gel electrophoresis. The prevalence of these piroplasms appeared to vary among elephant populations, with a higher prevalence recorded for Tsavo East elephants and Maasai Mara elephants at 18.8% (9/48) and 15% (3/20) respectively. Piroplams were not detected in Amboseli elephants (0/8) but prevalence was 7% (2/28) for Laikipia-Samburu elephants. This variation in piroplasm across populations was not statistically supported (χ^2^_3_ = 3.397, P = 0.334). Sixty-six elephant samples were from males and 38 were from females but prevalence was higher among females (18%) compared to males (11%) although this was not statistically significant (χ^2^_1_ = 0.68, P = 0.409). Six of the elephants (5.8%) were positive for *Anaplasma* or *Ehrlichia*. The prevalence of *Anaplasma* or *Ehrlichia* varied among elephant populations (χ^2^_3_ = 11.03, P = 0.012). A higher number of these were from Maasai Mara with a prevalence of 20% (4/20) followed by Amboseli with a prevalence of 12.5% (1/8) and TENP with a prevalence of 2% (1/48). There were no positive elephants in LSE (0/28).

A total of 41 adult *Amblyomma thollonii* ticks were identified. Thirty-four were males and 7 were females. Thirty-one of these ticks were from 20 elephants in the LSE, 5 ticks from 4 TENP elephants and 5 ticks from 3 AMBE elephants. A total of 11 adult *R*. *humeralis* ticks consisting of 10 male and 1 female collected from 5 elephants in TENP were identified. No ticks were collected or identified from Maasai Mara National Reserve. Out of all the *A*. *thollonii* ticks, gel electrophoresis results indicated that 51% (21/41) were positive for *Theileria* spp. or *Babesia* spp. whereas 19.5% (8/41) were positive for *Anaplasma* spp. or *Ehrlichia* spp. Out of all the *R*. *humeralis* ticks, 27% (3/11) were positive for piroplasms (*Theileria* spp. or *Babesia* spp.), and 18% (2/11) were positive for rickettsia (*Anaplasma* spp. or *Ehrlichia* spp.).

There was no association between the positive results based on gel electrophoresis of PCR products for *Theileria* or *Babesia* (χ^2^_1_ = 19.07, P<0.0001). Similarly, there was no association between positive results in ticks and their elephant hosts for *Anaplasma* or *Ehrlichia* (χ^2^_1_ = 8.782, P = 0.003). In fact, there was only a case in which the tick and the host elephant were positive for one group of pathogens; piroplasm, but all the elephants sampled were negative for pirosplasm and one was positive for the *Ehrlichia* spp. Overall, the prevalence of piroplasms and *Erhlichia* spp. in ticks was higher than that of their elephant hosts.

Sequence results and the GenBank BLAST search of 18S rRNA sequences revealed that elephants were infected with a single haplotype of *Babesia* (H1) which matched to a previously identified species of *Babesia* in elephants (Table 1). No similar *Babesia* was identified in any of the ticks picked from elephants. However, the elephant bont-tick, *A*. *thollonii* had 7 haplotypes of *Theileria* spp. consisting of two different species; 6 haplotypes, H2-H7 matched *Theileria bicornis* and a single haplotype H8 matched *Theileria* cf. *vellifera*. The piroplasm sequences from *A*. *thollonii* had a 94–100% match to the closest GenBank sample (Table 1). There were no clean sequences of piroplasm recovered from *R*. *humeralis*.

**Table 1:** GenBank Blast search results showing percent identity of the various piroplasm and rickettsia identified in ticks and their elephant hosts in Kenya. E-value for each haplotype– 0.00.

Haplotype ID	*Hosts*			Percent Identity	GenBank Accession Number	GenBank Species ID
	*Loxodonta africana*	*Ambyomma thollonii*	*Rhipicephalus humeralis*			
**H1**	12	0	0	100%	KU603425.1	*Babesia* spp.
**H2**	0	1	0	99%	MF536659.1	*Theileria bicornis*
**H3**	0	1	0	99%	MF536661.1	*Theileria bicornis*
**H4**	0	1	0	98%	MF536659.1	*Theileria bicornis*
**H5**	0	2	0	99%	MF536661.1	*Theileria bicornis*
**H6**	0	1	0	100%	MF536659.1	*Theileria bicornis*
**H7**	0	1	0	99%	MF536661.1	*Theileria bicornis*
**H8**	0	1	0	100%	GU733375.1	*Theileria velifera*
**H1**	1	4	1	94%	AB454077.1	*Ehrlichia* spp.
**H2**	0	1	0	93%	AB454077.1	*Ehrlichia* spp.
**H3**	0	1	1	93%	AB454077.1	*Ehrlichia* spp.
**H4**	0	1	0	93%	AB454077.1	*Ehrlichia* spp.
**H5**	2	0	0	94%	AB454077.1	*Ehrlichia* spp.

We did not detect *Anaplasma* in elephants or their tick ectoparasites. However, we detected five *Ehrlichia* haplotypes (H1 –H5) from both the elephants and their ticks. All the five haplotypes identified closely matched to a single haplotype of *Ehrlichia* species previously identified from Japanese deer (Table 1). Percent similarity was, however, low (93–94% sequence identity match) with known GenBank sequences. *Ehrlichia* haplotype H1 was shared among the elephant, and its ticks; *A*. *thollonii* and *R*. *humeralis*. Haplotype H2 and H4 occurred only in *A*. *thollonii* whereas haplotype 3 was present in both tick species (Table 1). Haplotype H5 was present only in the elephant (H5).

**Fig 2:**
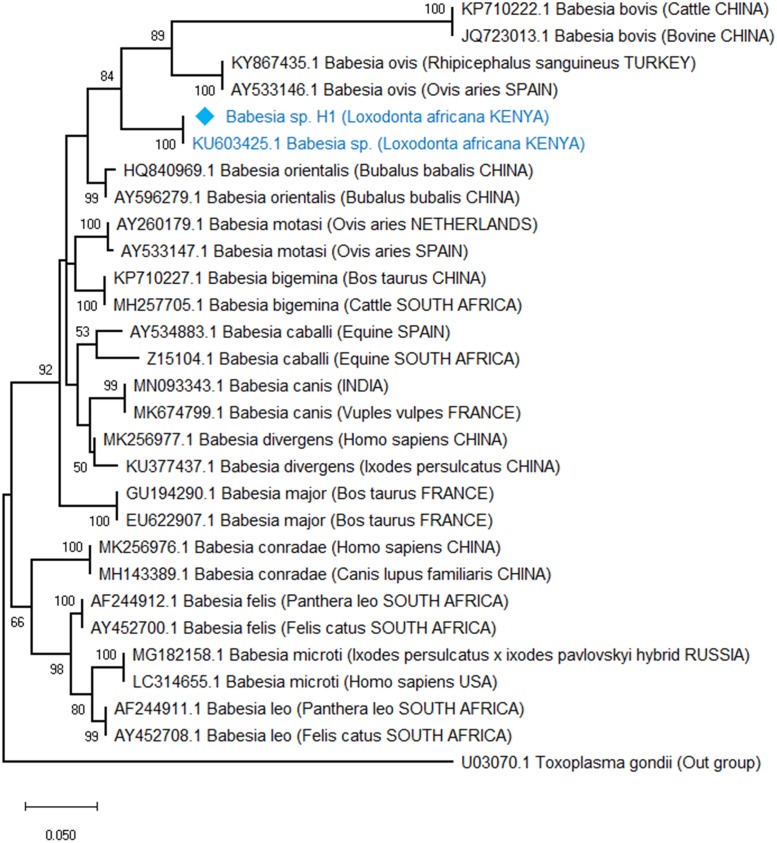
Phylogenetic relationship of the *Babesia* species isolated from the African elephant inferred from a 396 bp of the hypervariable V4 region of the 18S rRNA gene. The evolutionary relationships were inferred using the Maximum Likelihood method and the Kimura 2-parameter model with discrete Gamma distribution model for evolutionary rate differences among sites (5 categories (+*G*, parameter = 0.469) and a proportion of sites that are invariable (I = 0.346).

**Fig 3:**
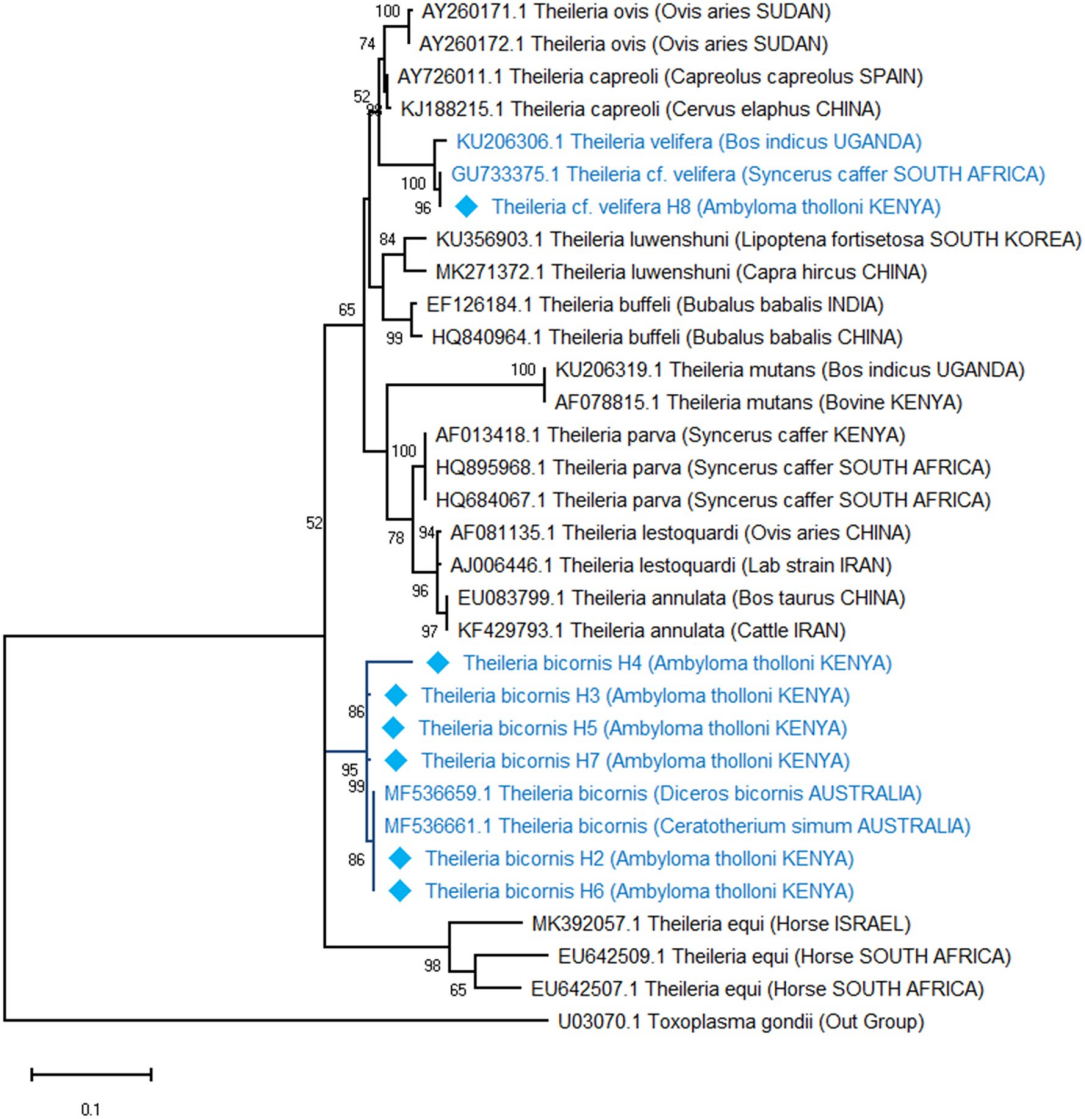
Phylogenetic relationship of *Theileria* species isolated from *Amblyomma thollonii* inferred from a 446 bp of the hypervariable V4 region of the 18S rRNA gene. The evolutionary relationships were inferred using the Maximum Likelihood method and the Kimura 2-parameter model with discrete Gamma distribution model for evolutionary rate differences among sites (5 categories (+*G*, parameter = 0.204) and a proportion of sites that are invariable (I = 0.471).

**Fig 4:**
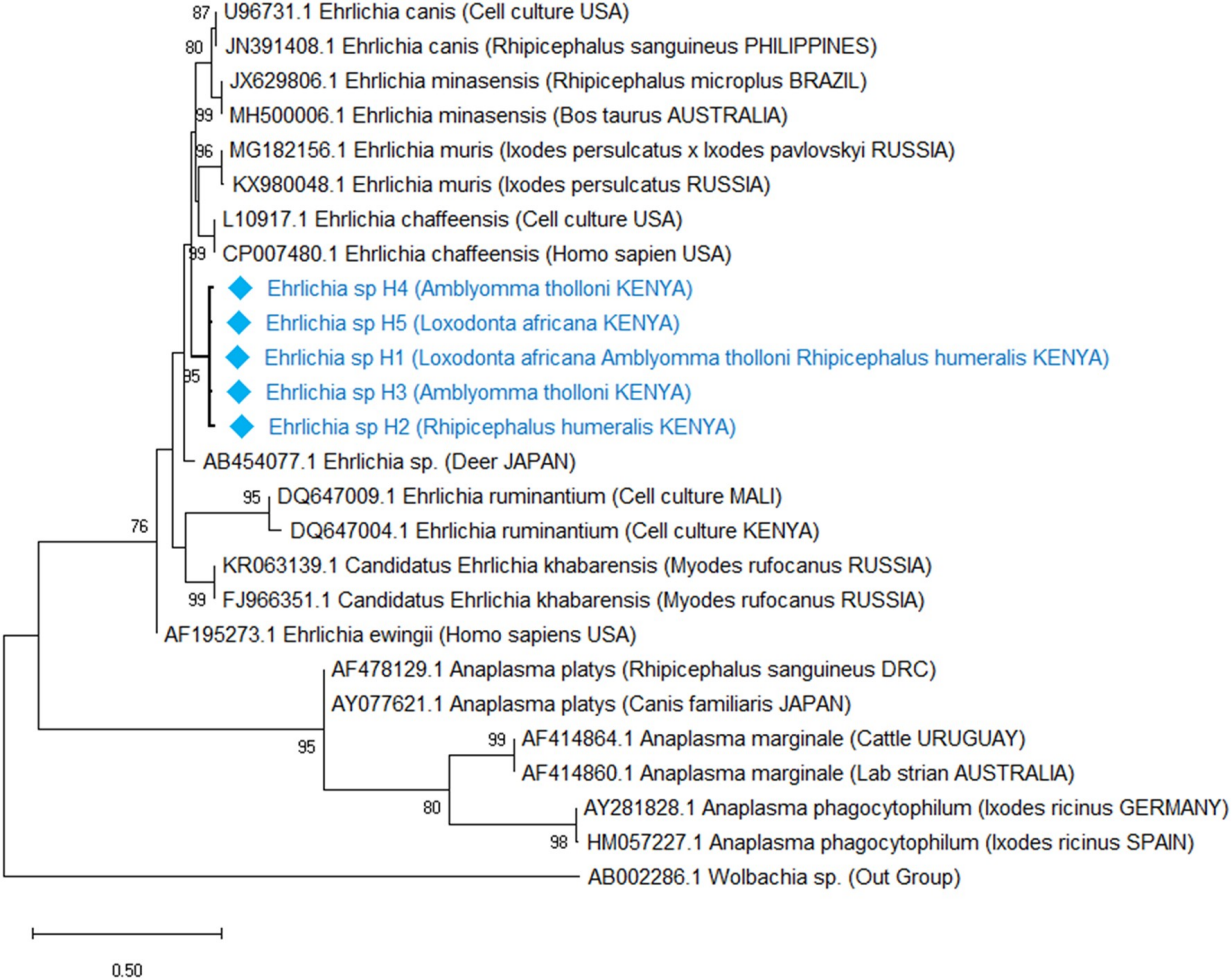
Phylogenetic relationship of *Ehrlichia* species inferred from 268 bp of the heat shock protein gene (groEL). The evolutionary relationships were inferred using the Maximum Likelihood method and the Tamura-3 parameter model with discrete Gamma distribution model for evolutionary rate differences among sites (5 categories (+*G*, parameter = 0.209).

Phylogenetic analyses revealed that the *Babesia* spp. detected in elephants is an unknown *Babesia* spp. previously detected in African elephants in Kenya with a 100% bootstrap support (Fig 2). *Theileria bicornis* and *Theileria* cf. *vellifera* haplotypes also clustered with sequence matches from GenBank with a 95% and 100% bootstrap support respectively (Fig 3). In contrast, *Ehrlichia* haplotypes did not cluster together with *Ehrlichia* spp. isolated from Japanese deer, which was its closest match from GenBank (Fig 4). However, all our haplotypes clustered together into a clade with 95% bootstrap support. Additionally, evolutionary distance analyses using the cantor model and assuming uniform rates of nucleotide evolution were consistent with both phylogenetic analyses and BLAST search matches (Table 1).

## Discussion

Pathogen identification and knowledge of pathogen infection patterns in a vector-host system is vital for understanding disease dynamics and emergence. In this study, we investigated patterns of infection by *Theileria*, *Babesia*, *Ehlirchia* and *Anaplasma* in elephants and their ticks and identified these pathogens using molecular genetic tools. The prevalence of piroplasm in Kenyan elephants at 13.5% was lower compared with infection patterns in other large African wild hosts. For example, the prevalence of piroplasm has been recorded to be 100% in common zebra, *Equus quagga*, Cape zebra, *Equus zebra* and Grevy’s zebra, *Equus grevyi* [30, 42, 43], 100% in free ranging spotted hyenas, *Crocuta crocuta* [44] and 92–100% in the African buffalo, *Syncerus caffer* [45]. However, the prevalence of piroplasm was comparable to that in south African black rhino, *Diceros bicornis* at 18.6% [46]. This was in contrasts to the prevalence of piroplasms in Black rhinos which had a prevalence of 43% [47], and white rhinos in Kenya with a prevalence of 66% [47]. In Kruger National Park South Africa, white rhinos, *Ceratotherium simum* had a piroplasm prevalence at 36.4% [48]. The major driver for variation in prevalence across populations observed in the literature could be related to individual host susceptibility and the species of piroplasm involved. In all cases above the higher rate of prevalence involved *Theileria* but not *Babesia*, the species we mostly identified in our study. It is also apparent that the rate of prevalence is lower for rhinos and elephants in the literature suggesting that 1). elephants and rhinos are less susceptible to infection from tick borne pathogens compared to other species, and or 2). The ticks that are competent vectors for these diseases do not invest in elephants and rhinos as preferred hosts.

The prevalence of *Anaplasma* and *Ehrlichia* was much lower than for piroplasms at 5.8% but with significant spatial variation in prevalence. This is comparable to a prevalence of 6% for *Ehrlichia* in African buffaloes from Chobe National Park and the Okavango Delta in Botswana [49] and 5% for *Ehrlichia ruminatum* at Kruger National Park South Africa [50]. This contrast a 0% rate detected in spotted hyenas and brown hyenas [44].

Prevalence of piroplasms (*Theileria* spp. or *Babesia* spp.) in *A*. *thollonii* ticks was 51% but was 27% in *R*. *humeralis* ticks. The observed prevalence was higher than recorded for most locations. The prevalence of piroplasms in adult ticks of two species of the genus *Rhipicephalus* (*R*. *evertsi evertsi* and *R*. *decoloratus*), from the Western Oromia region in Ethiopia revealed an overall prevalence of 4% (8/202) *Theileria buffeli*/*sergenti*/*orientalis*, and 2% (4/202) *Theileria ovis* [51]. However it was higher compared to a 2.7%; prevalence of T. parva infection in questing *R*. *appendiculatus* in the Tanga region, Tanzania [52].

The prevalence for *Anaplasma* spp. or *Ehrlichia* spp. was nearly identical in both *A*. *tholloni* and *R*. *humeralis* which was 19.5% and was 18% respectively. The prevalence was closely similar to a prevalence for *Anaplasma* and *Ehrlichia* infection rates of 16.4% from *Amblyomma* ticks parasitizing cattle and sheep in Ethiopia [53].

Overall, we observed that prevalence of *Erhlichia* and piroplasms was higher in tick vectors compared to the elephant. These findings contrasts those for the prevalence of *Theileria parva* between the tick, *Rhipicephalus appendiculatus* and their cattle host in Kenya where the infection rates for cattle compared to ticks was 43.5% and 2.3% in Limuru and 33% and 11.5% in Kakamega [54]. These differences may suggest strong immune selection of parasites by the elephant host than in the vector. This differential selection can be expressed as variation in pathogen load or DNA concentration between the vertebrate host and arthropod vector. Alternatively, these results may suggest that the reservoir vector for tickborne pathogens infecting elephants are tick or other species and elephants are a sink host.

Our results revealed infection of Kenyan elephants with a *Babesi*a spp. that is phylogenetically different from most of the commonly known species available in public genetic databases. This species was however identical to a species recently identified from Kenyan elephants and deposited in GenBank () by some of our colleagues. There are no other recent records of piroplasms in the elephant except for the historical information, on the description and identification of *Babesia loxodontis* (Rodhain, 1936) and later by Brocklesby and Campbell [18] from a sick elephant in Kenya using microscopy. The effect of Piroplasm infection on the health of the African elephant is not apparent but historical information suggests that it causes debilitation [18]. Moreover, Fowler and Mikota, [55], suggests that *Babesia* spp. infection is prevalent in Asian elephants where they are associated with weakness, fever, jaundice, constipation and haemoglobinuria.

We did not detect a *Babesia* species in the ticks detached from *Babesia*-infected elephants but rather, the ticks were infected with *Theileria* spp. It was of interest that the ticks did not share the *Theileria* spp. with the host elephant. Specifically, we identified *T*. *bicornis* in adult *A*. *thollonii* ticks that infested the elephants, but this piroplasm was not detected in elephants that hosted these ticks as well as the overall elephant population examined. Moreover, *T*. *bicornis* is an important piroplasm known to cause fatal infection in the endangered black rhinoceroses [5] but its tick vector is still unknown. Tick species that preferentially infest rhinoceros, *Dermacentor rhinocerinus* and *Amblyomma rhinocerotis* [5, 56] as well as *Rhipicephalus evertsi evertsi* [57] have been suspected as potential vectors. Our result is the first record of *T*. *bicornis* identified in a tick, and suggests *A*. *thollonii* as potential vector, though experiments on its vector competence are recommended. Further, absence of the parasite in elephants that hosted the infected ticks could imply that the parasite does not establish in the elephant. In addition, *A*. *thollonii* being a three-host tick, whose adult stage preferentially feeds on elephants, may have acquired the *T*. *bicornis* infection from rhinoceros in its earlier developmental stages (transtadial perpetuation) before infesting the elephant. *T*. *bicornis* is highly prevalent in the population of white and black rhinoceros in Kenya [47] where the populations of both rhinoceros and elephants are greatly overlapped.

Our results also showed that *A*. *thollonii* harbored *Theileria*. *cf*. *velifera*, which is seemingly maintained in the African buffalo (*Syncerus caffer*) [58]. In Kenya, *Theileria cf*. *velifera* has been detected in *A*. *eburneum* nymphs as well as *A*. *hebraeum* ticks infesting the African buffalo [59]. *T*. *velifera* is associated with benign theileriosis in cattle, sheep and goats [14] whereas its establishment in the elephant and the role of *A*. *thollonii* in the transmission of *T*. *velifera* are both unclear. However, it is likely that the infected young tick stages that feed on livestock may transmit *T*. *velifera* through transtadial perpetuation to the elephant.

We also identified *Ehrlichia* spp. in the African elephant and the elephant ticks, *A*. *tholloni* and *R*. *humeralis* but we did not detect *Anaplasma* spp. in either elephants or ticks. Ehrlichiosis, caused by multiple species including *E*. *chaffeensis*, *E*. *ewingii*, *E*. *canis*, and *E*. *ruminantium*, affects cattle, sheep, goats and dogs. *Ehrlichia* spp. has also been linked to Cowdriosis (heartwater) disease in the African elephant, [20]. However, the species in elephants have not been identified. The presence of identical and similar haplotypes of *Ehrlichia* in ticks and elephants suggest that *A*. *thollonii* and *R*. *humeralis* both with a wide host range are likely to be vectors for *Ehrlichia* spp. transmission between wild and domestic animals. Specifically transtadial perpetuation of *Ehrlichia* spp. by these three-host ticks, with tick larval and nymph stages of *A*. *thollonii* feeding on small domestic ruminants [14, 15] and adults feeding on elephants, shows that the elephant bont-tick and the elephant has a role in the epidemiology of Ehrlichiosis especially at the wildlife-livestock interface. According to MacKenzie and Norval [14], the heavy infestation of sheep by larvae and nymphs of *A*. *thollonii* were associated with frequent cases of Heartwater in the Zambezi valley of Zimbabwe. The tick, *R*. *humeralis* has been found in cattle and camels while among wild animals, it prefers African elephants and sometimes the rhinoceros and African buffalo [16, 17, 60]. Its distribution is restricted to southern Somalia, Eastern Kenya and Northern Tanzania [17]. In the current study, this tick was present only in the Tsavo elephants. There is no previous record on pathogens harbored by *R*. *humeralis* or disease association [17], thus our study provides the first record on the potential role of the tick species on the epidemiology of *Ehrlichia* spp.

## Conclusions

This is the first study of its kind conducted on elephant-tick-pathogen relationships in Kenya and provides a benchmark for evaluating elephant-tick-pathogen relationships elsewhere in the African continent where elephants occur. We describe for the first time molecular genetic identification of *Babesia* and *Ehrlichia* in the African elephant. The prevalence of *Babesia* and *Ehrlichia* in ticks was higher than that of their elephant hosts suggesting the reservoir status of the ticks for these pathogens. The pathogenic *T*. *bicornis* has never been isolated from any tick species. From our studies, we recommend further studies to understands the drivers for variation in prevalence of *Theileria*, *Babesia* and *Ehrlichia* in African elephants and their ticks and an investigation of the vector competence of *A*. *thollonii* for *Theileria bicornis*, an important pathogen for rhinos.

## Supporting information

S1 Text (TXT)
